# The challenges of time for studies on the population effects of the COVID‐19 pandemic on perinatal outcomes

**DOI:** 10.1111/ppe.12958

**Published:** 2023-01-30

**Authors:** Jennifer Zeitlin

**Affiliations:** ^1^ Obstetrical, Perinatal and Pediatric Epidemiology Research Team UMR 1153 Inserm and Université Paris‐Cité Paris France

The temporal dimension of research on the COVID‐19 pandemic and pregnancy outcomes poses multiple challenges for perinatal epidemiologists. This issue of *Paediatric and Perinatal Epidemiology* features two studies and associated commentaries^1^, ^2^, ^3^, ^4^ that investigate population‐level changes in preterm birth and in vitro fertilisation (IVF) births related to the COVID‐19 pandemic and speak to two of these challenges: using appropriate methods for time trend analyses, that is, time series as opposed to before‐after designs, and appropriate denominators to capture time‐varying exposures and potential changes in the characteristics of the childbearing population, that is, conception versus birth cohort designs.

Both methodological approaches differ from those in the usual perinatal epidemiology toolbox, and their increasing use in COVID‐19 research holds more general lessons for research in our field. For instance, an assumption in before‐after designs (termed the ‘stacked calendar’ approach by Bruckner and Gemmill^4^) is that it is possible to control for time trends by adjusting for changes in important population characteristics such as maternal age, body mass index, educational level or smoking. However, for preterm birth rates, this approach does not explain all changes over time, as seen by widely contrasting contemporaneous preterm birth trends in high‐income countries despite similar evolution of these sociodemographic characteristics.^5^ Inaccurate modelling of time trends may be one explanation for the contradictory results that fuel continued debate about the existence, magnitude, and reasons for preterm birth rate decreases during the pandemic. Other issues, raised in the commentaries, include the management of outliers and derivation of confidence intervals when evaluating time series.^4^

The use of conception versus birth cohorts, a topic addressed by all the studies in this issue, also challenges perinatal epidemiologists to refine our concepts of the population at risk. A pandemic is a major disruption that calls into question the underlying assumptions of stable fertility trends and childbearing patterns on which we silently rely when using birth cohort denominators. While these assumptions likely hold over the limited temporal lag between most numerators (i.e. preterm births or stillbirths) and their birth cohort denominators (all births, mostly at term), it is possible that they distort some analyses in systematic ways. Despite the vigorous debate in our field about using a fetus‐at‐risk denominator for the analysis of stillbirth risks,^6^ for instance, it is telling that the ‘at risk’ denominator—in a temporal sense—never strictly captures the true population at risk. Because most stillbirths occur in the 5th to the 7th months of pregnancy, analyses over a one‐year period approximate the at‐risk population for stillbirths occurring at the year's end with term births at the beginning of the year.

The pandemic has highlighted other time challenges, including the possibility of greater left truncation bias due to pandemic‐related early losses, or immortal time, when assessing risks associated with infection at delivery only. Both of these points, raised in previous *Paediatric and Perinatal Epidemiology* commentaries,^7^, ^8^, ^9^ complicate the interpretation of many studies on the pandemic, but they also lead to broader questions about the suitable management of time in other perinatal epidemiology research.

Time is also a major challenge for population research on COVID‐19 because of lags in data availability. Population databases are clunky; data are transferred with delay to a central repository from local civil registration systems or clinical registers or hospital databases, then compiled, checked, and possibly linked to other sources. Because of this, most population data are only available with substantial lags—that is, finalised data from 2020 became available in many countries only in late 2021 or in 2022.^10^ The need to assess conception cohorts occasions more delay, as the denominators for key events—stillbirths and preterm births—will occur the following year (as described in the limitations in the study by Lisonkova and colleagues^1^). This delay in data availability is reflected in the publication patterns of studies on COVID‐19 and pregnancy in clinical versus epidemiology journals from 2020 to 2022 (Figure 1). These limits of population birth data have constrained epidemiological research on the current pandemic and constitute a warning in our field to prepare for future pandemics by improving the availability and reactivity of population data.

**FIGURE 1:**
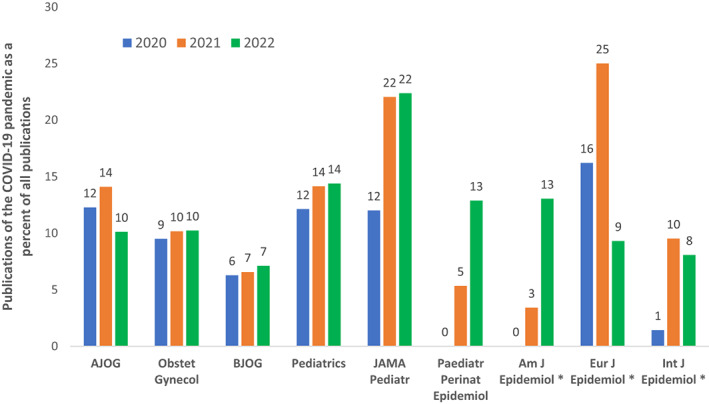
Publications on COVID‐19 from 2020 to 2022 as a per cent of all publications in obstetrics and paediatric journals and of publications on obstetrics and paediatrics in epidemiological journals. *Notes:* * only publications related to obstetrics and paediatrics (see below for definition). *Search terms*. (1) publications over the period in a given journal: (‘journal name’[Journal]) AND ((‘2020/01/01’[Date—Publication]: ‘3000’[Date—Publication])); (2) publications on COVID‐19: AND (covid OR SARS‐CoV‐2 or coronavirus); (3) publications related to obstetrics and paediatrics (for epidemiology journals only): AND (pregnancy OR obstetric* OR preterm OR stillbirth OR birth OR neonat* OR newborn OR infant OR child* OR paediatric* OR paediatric*). *Denominators*: That is, the number of total articles per year presented as Journal name (2020, 2021, 2022): AJOG (790,780,830); Obstet Gynecol (590,423,381); BJOG (621,595,492); Paediatrics (866,813,695) JAMA Pediatr (408,431,389); Paediatr Perinat Epidemiol (138,131,132); Am J Epidemiol (52,88,69); Eur J Epidemiol (37,40,43); Int J Epidemiol (70,105,161). *Date of search*: January 12, 2023.

These time lags give rise to a more basic challenge—identifying the purpose of research on the effects of the pandemic with a two‐ or three‐year delay. The relevance of the lockdowns of 2020 and of the variant of the virus circulating in 2020 to the day‐to‐day management of the pandemic in 2023 is not obvious. Objectives of research could be to evaluate the impact of policies in 2020 to inform future lockdowns—as related to the closure of IVF services during the 2020 lockdowns in the study by Lisonkova et al.^1^—or to shed light on the causes and risk factors of adverse perinatal outcomes by using this highly unusual event as an experiment. By assessing conception cohorts, Margerison and colleagues^2^ seek to explore whether population shocks might have a greater impact when they occur earlier in gestation. It is vitally important to clarify specific objectives because this determines the methodology and the framework of the analysis as well as the assessment of its limitations. This may be particularly important when using the pandemic as an ‘experiment’, since the simultaneous change of so many health systems, lifestyle, and health conditions in non‐monotonic ways renders it an imperfect instrument.^11^

A final, but often underappreciated, reason for this research is to gather knowledge and ideas to improve population research on maternal and newborn outcomes during infectious disease pandemics. This includes thoughts about priority research questions, optimal designs and methods, and timeliness and availability of data. For instance, our understanding of preterm birth decreases in 2020 is limited by poor ascertainment of spontaneous versus indicated preterm birth in many population databases. Other ideas include developing databases to register early losses to allow investigation of left truncation bias or creating protocols to facilitate rapid international exchange and analysis of data. This is the time to get a head start on the future.

## About the author

**Jennifer Zeitlin** is a tenured Research Director at INSERM (French National Institute of Health and Medical Research) in the Obstetrical, Perinatal and Pediatric Epidemiology Research Team, U1153, Paris. Her research focuses on the impact of the organisation and quality of medical care on maternal and newborn health. She leads European projects on perinatal health indicators (Euro‐Peristat) and very preterm birth cohorts. She also works on quality of care and disparities with a research group at the Department of Population Health Science and Policy, Icahn School of Medicine at Mount Sinai, New York, where she is an adjunct professor. She is deputy editor at *Paediatric and Perinatal Epidemiology*.
